# Development of Recombinant PLC-Zeta Protein as a Therapeutic Intervention for the Clinical Treatment of Oocyte Activation Failure

**DOI:** 10.3390/biomedicines12061183

**Published:** 2024-05-27

**Authors:** Alaaeldin Saleh, Angelos Thanassoulas, Elnur Aliyev, Karl Swann, Azza Naija, Huseyin C. Yalcin, F. Anthony Lai, Michail Nomikos

**Affiliations:** 1 Department of Basic Medical Sciences, College of Medicine, QU Health, Qatar University, Doha 2713, Qatar; 2 School of Biosciences, Cardiff University, Cardiff CF10 3AX, UK; 3 Biomedical Research Center, Qatar University, Doha 2713, Qatarhyalcin@qu.edu.qa (H.C.Y.); 4 Department of Biomedical Sciences, College of Health Sciences, QU Health, Qatar University, Doha 2713, Qatar

**Keywords:** male infertility, phospholipase C, PLC-zeta, sperm, IVF, ICSI, fertilization, OAF

## Abstract

The sperm-specific phospholipase C zeta (PLCζ) protein is widely considered as the predominant physiological stimulus for initiating the Ca^2+^ release responsible for oocyte activation during mammalian fertilization. The increasing number of genetic and clinical reports that directly link PLCζ defects and/or deficiencies with oocyte activation failure (OAF) necessitates the use of a powerful therapeutic intervention to overcome such cases of male factor infertility. Currently, in vitro fertilization (IVF) clinics treat OAF cases after intracytoplasmic sperm injection (ICSI) with Ca^2+^ ionophores. Despite their successful use, such chemical agents are unable to trigger the physiological pattern of Ca^2+^ oscillations. Moreover, the safety of these ionophores is not yet fully established. We have previously demonstrated that recombinant PLCζ protein can be successfully used to rescue failed oocyte activation, resulting in efficient blastocyst formation. Herein, we produced a maltose binding protein (MBP)-tagged recombinant human PLCζ protein capable of inducing Ca^2+^ oscillations in mouse oocytes similar to those observed at fertilization. Circular dichroism (CD) experiments revealed a stable, well-folded protein with a high helical content. Moreover, the recombinant protein could retain its enzymatic properties for at least up to 90 days after storage at −80 °C. Finally, a chick embryo model was employed and revealed that exposure of fertilized chicken eggs to MBP-PLCζ did not alter the embryonic viability when compared to the control, giving a first indication of its safety. Our data support the potential use of the MBP-PLCζ recombinant protein as an effective therapeutic tool but further studies are required prior to its use in a clinical setting.

## 1. Introduction

Mammalian fertilization is a multi-step process, which is initiated straight after sperm–egg fusion and followed by an acute rise in the intracellular levels of free Ca^2+^ concentration [Ca^2+^] within the egg cytoplasm [1,2,3]. In mammals, this increase in [Ca^2+^] has the form of repetitive, long-lasting Ca^2+^ oscillations, which are responsible for the activation of a number of signaling pathways leading to cortical granule exocytosis, resumption of meiosis, pronuclear formation and eventually to early embryogenesis [4,5,6]. It is now clearly apparent that the sperm-specific phospholipase C zeta (PLCζ) protein is the key factor for initiating the Ca^2+^ oscillations that are sufficient for the completion of all the aforementioned events that mark egg activation [7,8,9,10,11,12,13,14]. Upon initiation of the acrosome reaction, the PLCζ protein diffuses from the sperm head into the ooplasm and hydrolyzes its membrane-bound substrate, phosphatidylinositol 4,5-bisphosphate (PIP_2_), to two second messengers, inositol trisphosphate (IP_3_) and diacylglycerol (DAG). IP_3_ then binds to the IP_3_ receptors (IP_3_R) located at the endoplasmic reticulum (ER), resulting in Ca^2+^ release into the cytoplasm of the oocyte in the form of repetitive and persistent oscillations, which last until the formation of the pronuclei [3]. 

The sperm PLCζ protein is ~70 KDa in size and consists of four distinct domains. Two pairs of EF hand motifs reside at the N-terminus, followed by the X and Y catalytic domains responsible for the hydrolysis of PIP_2_ and the C-terminal C2 domain [3]. The X and Y catalytic domains are separated by an unstructured region that contains a number of positively charged amino acids, the XY linker region. Despite its small size compared to the somatic PLC isoforms, sperm PLCζ has a distinctively unique potency in triggering Ca^2+^ oscillations within the fertilizing egg that is attributed to its novel molecular characteristics, arising from the essential role of its domains [3,15].

For the last several years, different genetic and clinical studies have reported a number of PLCζ mutations identified in individuals with oocyte activation deficiencies, resulting in fertilization failure [3,16]. These mutations can either lead specifically to reduced expression levels or inactive PLCζ protein in the sperm of these patients, resulting in oocyte activation failure (OAF) [9,10,11,12,13,14]. The strong correlation of all the identified PLCζ mutations with OAF and eventually male factor infertility is a powerful indication that, at least in humans, PLCζ is the primary oocyte-activating factor responsible for initiating the Ca^2+^ oscillations that “kick start” mammalian fertilization.

Nowadays, in vitro fertilization (IVF) clinics routinely use state-of-the-art techniques like intracytoplasmic sperm injection (ICSI) to efficiently overcome many severe cases of male infertility arising from defects in sperm morphology or motility [16]. However, in order to overcome cases of OAF, IVF clinics employ artificially mediated Ca^2+^ release and Ca^2+^ ionophores, like ionomycin or calcimycin, to artificially activate the oocyte and overcome unexplained fertilization failure after repeatedly failed ICSI cycles [13]. 

Recent systematic reviews and meta-analysis studies have suggested that the use of Ca^2+^ ionophores significantly improved fertilization and implantation rates in ICSI as well as offspring safety [17]. On the other hand, sibling oocyte controlled studies suggest that ionophores may be ineffective in activating development [18]. In contrast to the repetitive transients of Ca^2+^ observed at physiological fertilization, Ca^2+^ ionophores only cause a single transient of Ca^2+^. In addition to their unclear effectiveness, there are still many concerns associated with the use of Ca^2+^ ionophores to artificially activate the oocyte regarding long-term safety and potential future transgenerational effects. Since the long-term safety of Ca^2+^ ionophores is not yet well established, several studies have raised concerns about the teratogenic, cytotoxic and epigenetic effects of ionophores on the embryo [16,19,20]. 

Therefore, the use of an endogenous agent like PLCζ would represent the ideal intervention to treat cases of OAF. We have previously reported that the microinjection of human recombinant PLCζ yielded higher blastocyst development rates than Ca^2+^ ionophore treatment [21]. In vitro production of active, stable, purified versions of human recombinant PLCζ protein that retain their activity for long periods would be a major step forward in enabling its routine use by IVF clinics as an alternative method to clinically treat cases of oocyte activation failure and/or deficiency. In this study, we have generated active recombinant human MBP-tagged PLCζ protein and we investigate its Ca^2+^ oscillation-inducing activity in mouse eggs, its biophysical characteristics and its stability over time. Finally, we employ a chicken embryo model to provide an initial indication regarding its safety.

## 2. Materials and Methods

### 2.1. Recombinant Protein Expression and Purification

*Escherichia coli* (BL21CodonPlus(DE3)-RILP; Stratagene, La Jolla, CA, USA) cells were transformed with the pETMM41-PLCζ construct [22] and cultured at 37 °C to an OD600 of 0.6. Protein expression was then induced at 16 °C for 18 h by adding 0.1 mM isopropyl β-D-thiogalactopyranoside (IPTG; Thermo Scientific, Vilnius, Lithuania). Induced cells were then harvested by centrifugation at 6000× *g* for 10 min at 4 °C and resuspended in ice-cold amylose column lysis buffer [20 mM Tris–HCl (pH 7.4), 250 mM NaCl, 1 mM EDTA, 10 mM Maltose, 1 mg/mL lysozyme and EDTA-free Protease Inhibitor Tablets (Roche, Indianapolis, IN, USA)]. The resuspended cells were sonicated four times for 15 s on ice. After 30 min of centrifugation at 20,000× *g* at 4 °C, the soluble MBP-tagged fusion proteins were purified by affinity chromatography using an amylose resin column following standard protocols (New England Biolabs, Ipswich, MA, USA). Eluted proteins were then dialyzed in Phosphate-Buffered Saline (PBS) and concentrated using 30 k MWCO centrifugal concentrators (Sartorius; Stonehouse, Gloucestershire, UK).

### 2.2. Recombinant Protein Microinjection in Mouse Eggs

Mouse eggs were collected from super-ovulated CD1 female mice as described previously [23]. Eggs were maintained in M2 medium (Sigma–Merck, Saint Louis, MO, USA) and microinjected with a 1:1 mixture of 1 mM Oregon Green BAPTA dextran (Thermo Scientific, Vilnius, Lithuania), and MBP-PLCζ recombinant protein. Microinjections were carried out as described by Fitzharris et al. [24]. Fluorescence recordings were from eggs in HKSOM medium on a heated stage of a Nikon Eclipse inverted fluorescence microscope as described by Ikie-Eshalomi et al. [23]. Ca^2+^ oscillations started immediately after the injection of recombinant protein and so records commence with oscillations from the start.

### 2.3. Circular Dichroism (CD)

CD measurements were carried out using the Jasco J-1100 spectrophotometer equipped with a Jasco PTC-514 Peltier thermostatted cell holder (Jasco, Tokyo, Japan) using a quartz cuvette with a path length of 0.1 cm (Hellma, Müllheim, Germany). MBP-tagged recombinant PLCζ protein was dissolved in PBS buffer and the concentration was calculated based on the absorbance at 280 nm as previously described [22]. Far-UV CD spectra were collected in the wavelength range of 260–180 nm with a scan speed of 50 nm/min, using a sample concentration of 1.8 mg/mL to investigate the secondary structure of the MBP-PLCζ. A total of 8 accumulations were recorded and the raw CD data (mdeg) were subtracted from the buffer spectra (PBS). Spectra for the denatured protein were obtained at 90 °C. Thermal melting profiles were collected by measuring the molar ellipticity changes at 222 nm in the temperature range 20–90 °C at a rate of 1 °C per minute. The melting temperature was estimated using OriginPro software from non-linear curve fitting assuming a two-state folded-to-unfolded kinetic model, as previously described [25].

### 2.4. PLC Activity Assay

The PIP_2_ hydrolytic activity of MBP-PLCζ was analyzed as previously described [22,26]. The final PIP_2_ concentration in the reaction mixture was 220 mM, containing 0.05 mCi of [^3^H]PIP_2_. The hydrolysis assay was measured at different time points (0, 1, 7, 14, 30, 60, 90 days), as well as different temperature conditions (4 °C, −20 °C and −80 °C). To examine the Ca^2+^ dependence of PLC enzymatic activity, the Ca^2+^ buffers were prepared by EGTA/CaCl_2_ admixtures as previously described [26]. K*_m_* and EC_50_ values of Ca^2+^ dependence for PIP_2_ hydrolysis for the MBP-tagged PLCζ recombinant protein were determined by non-linear regression analysis (GraphPad Prism 9.5.1). 

### 2.5. Embryonic Chick Culture and in Ovo Treatment

Fertilized white Leghorn chicken eggs (*Gallus gallus*), obtained from a local poultry farm (Arab-Qatari Poultry Farm, Doha, Qatar), were directly incubated at 37.5 °C using a GQF 1500 Digital Sportsman incubator with a fully filled water tank to provide 60% relative humidity. The eggs were divided into three groups: Group 1 (*n* = 55): non-injected “control”; Group 2 (*n* = 55): injected with 200 µL of sterile PBS “sham control”; and Group 3 (*n* = 55): injected with 1 µg of MBP-PLCζ recombinant protein suspended in PBS “treatment”. On embryonic day (ED) 0, the surfaces of egg shells were disinfected with 70% ethanol and a small hole was drilled at the blunt end of the egg. The embryos were treated by injection into the yolk using a Hamilton syringe. Then, the hole was sealed with PVC tape. The eggs were candled daily to inspect the viability of the embryos. Infertile eggs and dead embryos were removed from the incubator and inspected for abnormalities. The experiment was terminated at ED10. The viable eggs were cracked and inspected visually for vascularization, and developmental abnormalities. The experiment was replicated 3 times. A Kaplan–Meier curve was used to indicate the survival of treated and control groups. GraphPad prism was used to estimate the statistical significance between groups. *p*-values of ≤0.05 were considered statistically significant. 

## 3. Results

### 3.1. Recombinant Human MBP-Tagged PLCζ Protein Has Potent Ca^2+^ Oscillation-Inducing Activity

**Figure 1:**
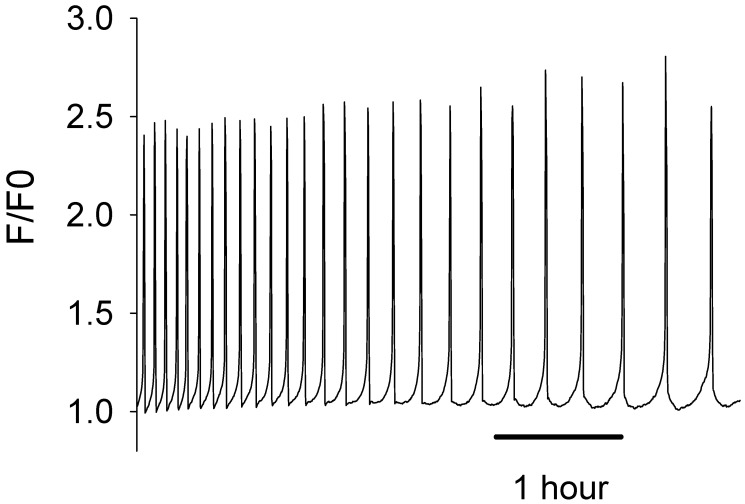
Microinjection of recombinant human MBP-PLCζ protein in mouse eggs triggers Ca^2+^ oscillations similar to those observed following microinjection of native sperm extracts. The traces show an example of a mouse egg injected with MBP-PLC*ζ*. The cytosolic Ca^2+^ is shown as the self-ratio of fluorescence (F/F0) for OGBD. In total, 22 eggs were injected with ~0.05 mg/mL protein. There was a mean of 10.5 spikes per hour (S.E.M. = 1.76).

The production of active recombinant human PLCζ protein over the last two decades has been very challenging. Our earlier efforts to generate either untagged or only hexahistidine (6xHis) tagged versions of recombinant human PLCζ protein using a bacterial expression system have proven unsuccessful, yielding either an insoluble and/or non-functional PLCζ protein. However, we have previously demonstrated that both NusA and MBP are extremely effective fusion tags for human PLCζ protein bacterial expression, facilitating the production of soluble and active recombinant PLCζ protein [22,27]. Moreover, as we previously reported, our comparative experiments with NusA and MBP tags showed that MBP is a more effective protein fusion partner for PLCζ [22]. As shown in Figure 1, microinjection of recombinant human MBP-PLCζ into mouse eggs at a concentration of 0.05 mg/mL revealed that it possesses potent Ca^2+^ oscillation-inducing activity, triggering distinctive Ca^2+^ oscillations mimicking those observed after microinjection of native sperm extracts. The chosen concentration of recombinant PLCζ protein for microinjection experiments in mouse eggs has been used in our previous studies [22,27], following a number of optimization experiments, and appeared to be effective in inducing Ca^2+^ oscillations in all eggs injected. 

Furthermore, all eggs that were injected with the recombinant human MBP-PLCζ protein formed second polar bodies, which is the first sign of egg activation. We did not follow these eggs for a long development time, as during imaging they were exposed to light, and this affects development. Developmental studies have been performed in separate experiments. We have demonstrated in our previous studies that once PLCζ induces the Ca^2+^ oscillations it can trigger development to the blastocyst stage [27]. 

### 3.2. CD Spectra and Protein Stability of MBP-PLCζ Recombinant Protein

**Figure 2:**
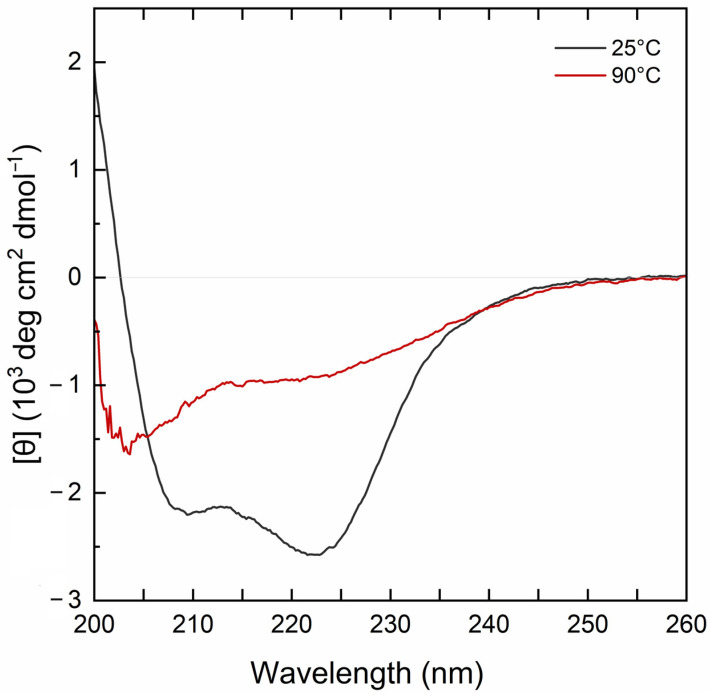
CD analysis of the human MBP-PLCζ recombinant protein. Normalized far-UV CD spectra for recombinant human MBP-PLCζ protein at 25 °C (black) and 90 °C (red).

**Figure 3:**
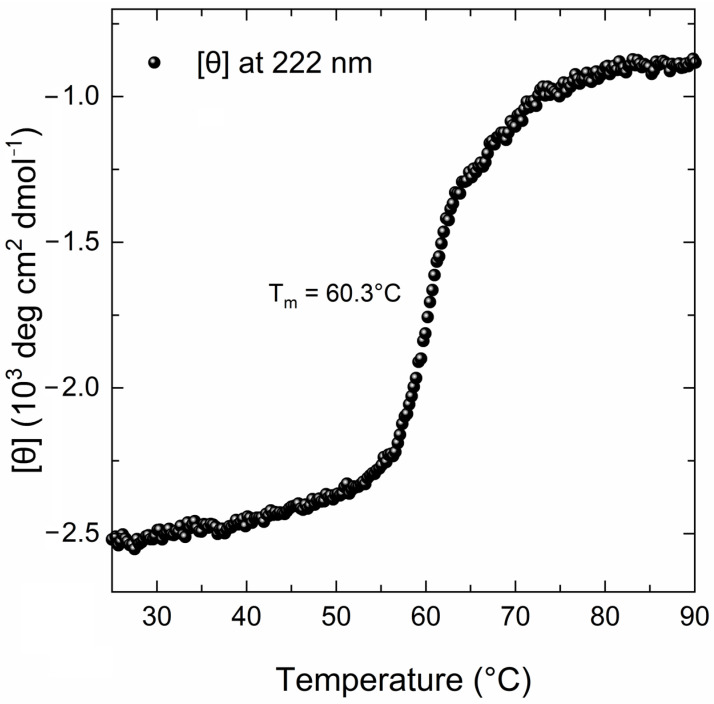
Thermal denaturation curve of the human MBP-PLCζ recombinant protein by CD spectroscopy. The CD data are plotted as molar residue ellipticity ([θ]) at 222 nm versus temperature, as recorded by heating the sample from 25 °C to 90 °C at a constant rate of 1 °C/min.

The far-UV CD spectrum of the MBP-PLCζ protein at 25 °C demonstrated a well-folded protein structure (Figure 2, black line). The CD spectrum displayed two characteristic minima at 222 nm and 208 nm, suggesting a high helical content for the MBP-PLCζ protein. Heating the sample to 90 °C led to thermal unfolding of the protein, which adopted a structure resembling a random-coil conformation with a characteristic negative peak at 200–205 nm (Figure 2, red line). However, some secondary structure elements were retained in the final structure, as the CD signal in the 220–225 nm region was approximately 30% that of the protein at 25 °C. The thermal melting profile of MBP-PLCζ, monitored at 222 nm, revealed that changes at the secondary structure level begin at 55 °C (Figure 3, black circles), while the melting temperature of the thermal transition is T_m_ = 60.3 °C. At 70 °C, the thermal transition is fully completed. The MBP-PLCζ thermal transition is not reversible, since no further change in the spectrum is observed when the protein is cooled back to 25 °C and visual inspection of the sample revealed the formation of large aggregates.

### 3.3. Recombinant Human MBP-PLCζ Protein Retains Its Enzymatic Properties Following 90-Day Period at −80 °C

**Figure 4:**
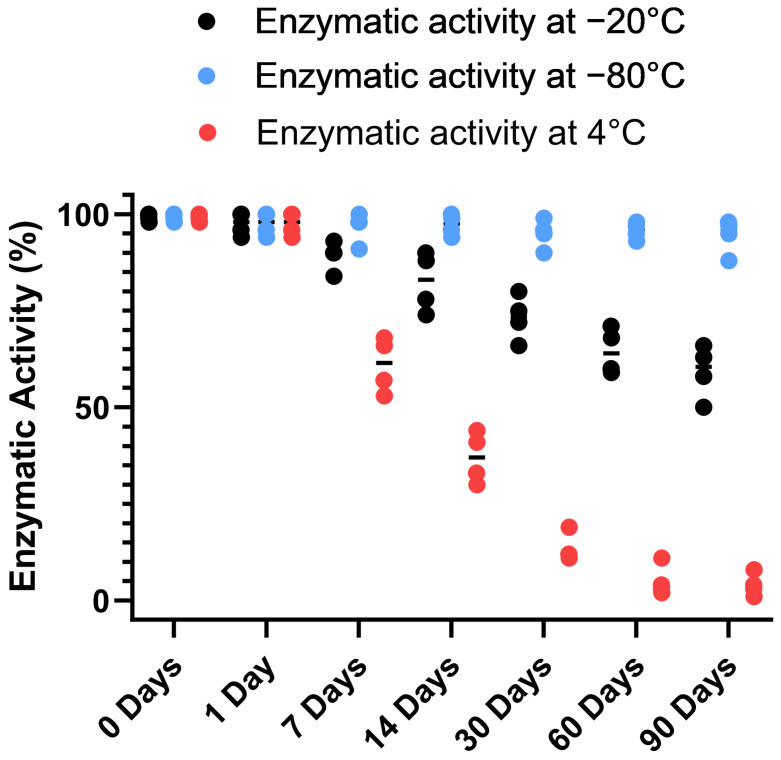
Analysis of recombinant MBP-PLCζ enzymatic activity over time and under different storage conditions. Normalized in vitro enzyme specific activity of recombinant human MBP-PLCζ protein following a 90-day storage period at 4 °C (red), −20 °C (black) and −80 °C (blue). PIP_2_ hydrolysis enzyme activity of recombinant human MBP-PLCζ protein was obtained using the standard [^3^H]PIP_2_ hydrolysis assay.

**Figure 5:**
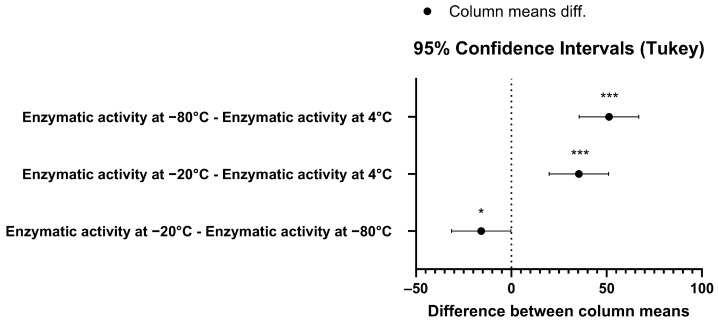
Pair-wise comparison confidence intervals plot using Tukey’s test for the enzymatic activity data at 95% family-wise confidence level. The *p*-value for the −80°C–4°C pair was *p* = 0.047 (*), while for the other two pairs the *p*-value was *p* < 0.001 (***).

**Figure 6:**
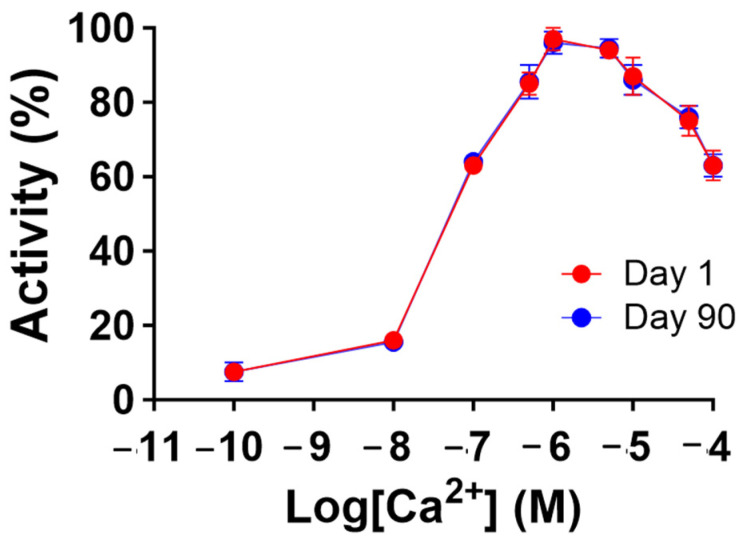
Recombinant human MBP-PLCζ protein retains its Ca^2+^ sensitivity following 90-day storage at −80 °C. Effect of various Ca^2+^ concentrations on the normalized enzymatic activity of recombinant human MBP-PLCζ protein following a 90-day storage period at −80 °C. For these assays, values are ±SEM (*n* = 4); *t*-test analysis comparing day 1 to day 90 responses failed to detect any statistically significant differences for the K*m* and EC_50_ values of the protein samples (GraphPad, Prism 9.5.1).

To gain further insight into the stability of the recombinant human MBP-PLCζ protein, we monitored its in vitro enzymatic activity over time (0 to 90 days) and at different storage conditions, including storage at 4, −20 and −80 °C. The PIP_2_ hydrolysis assay was employed, using the optimized conditions, as we have previously described [26,28,29]. As shown in Figure 4, storing the MBP-PLCζ protein for extended periods at 4 °C and −20 °C resulted in a significant reduction of its enzymatic activity. More specifically, storage at −20 °C for 90 days led to a remarkable reduction in the specific activity of MBP-PLCζ to hydrolyze PIP_2_, while storage at 4 °C for 90 days led to an almost complete loss of its enzymatic activity. In contrast, storage of the protein at −80 °C for the same 90-day period did not affect its enzymatic activity. To test whether there was significant variation in means among the enzymatic activities at different storage conditions, we performed a nested one-way ANOVA followed by the post hoc Tukey HSD (Honestly Significant Difference) test calculator. The results are presented in Figure 5 and clearly demonstrate that storage at −80 °C is the most efficient temperature for the recombinant MBP-PLCζ protein to retain its enzymatic activity among all groups tested. Moreover, the protein stored at −80 °C retained all its enzymatic properties, including Ca^2+^ sensitivity and Michaelis–Menten constant *Km* (Figure 6, Table 1). 

### 3.4. Exposure of Recombinant MBP-PLCζ Protein Did Not Affect the Embryonic Viability of Chicken Embryos

**Figure 7:**
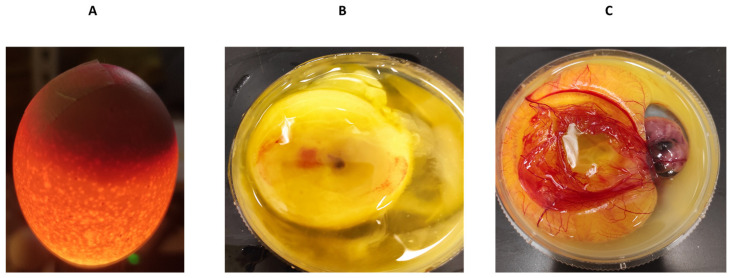
Examination of chicken embryos by candling technique. The candling technique was used to identify infertile/dead eggs and observe the vascularization and growth of viable embryos (**A**). To confirm the findings, eggs were cracked into a 100 mm dish to identify the dead (**B**) or the viable (**C**) embryos.

**Figure 8:**
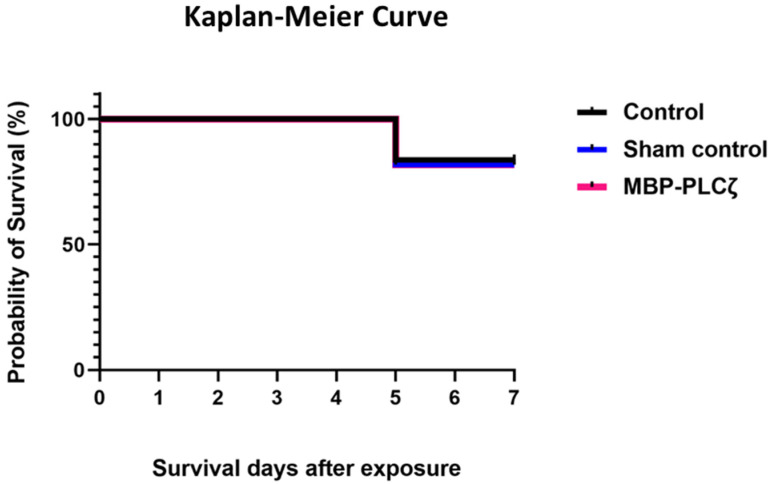
Survival analysis of chick embryos exposed to MBP-PLCζ recombinant protein and their matched controls. Exposure of the embryos to 1 µg of MBP-PLCζ had no effect on the survival probability in comparison to the control groups. The log rank test was performed to compare the survival rates and analysis, showing that there was no statistical difference at α = 0.05 between the two groups and the control (non-significant, *p* > 0.05).

To obtain an initial indication and investigate any potential cytotoxic effects of recombinant human MBP-PLCζ protein, we employed a chick embryo model. The chick embryo model provides a fast, cost-effective and reproducible method for investigating the effect of various test compounds on developmental toxicity. The combination of ease of handling, rapid development, accessibility, ethical considerations, homology to mammalian systems and experimental flexibility makes chicken embryos an attractive first model for such studies. The effect of MBP-PLCζ recombinant protein on the embryonic viability was investigated over a period of 7 days. The eggs were divided into three groups, where 55 eggs served as control (non-injected), 55 eggs were exposed to PBS (sham) and 55 embryos were exposed to 1 µg of recombinant MBP-PLCζ protein (treatment) (Table 2). The eggs were examined daily using candling to distinguish live from dead embryos (Figure 7). At ED5, there was a slight decrease in the survival of all three groups (between 15–18%). This decrease in the survival rate (SR) may be attributed to the critical nature of the first developmental embryonic days of chickens. As shown in the Kaplan–Meier survival curve, the rate of survival did not differ between the treatment, control and sham groups until the endpoint of the study (Figure 8). Our data suggested that exposure to 1 µg of recombinant human MBP-PLCζ protein did not affect the embryonic viability of chick embryos.

## 4. Discussion

Over the last two decades, a substantial amount of evidence supports the notion that the smallest identified mammalian PLC isoform, PLCζ, with the most basic domain organization is the physiological stimulus that triggers intracellular Ca^2+^ oscillations during the first steps of mammalian fertilization [3,15,16,30]. Compared to the other somatic PLC isoforms, PLCζ exhibits a uniquely supreme potency in generating Ca^2+^ oscillations within the fertilizing eggs that are sufficient to induce egg activation and early embryonic development [3]. It is well established that PLCζ is delivered by the fertilizing sperm into the egg cytoplasm, triggering the Ca^2+^ oscillations via the InsP_3_ signaling pathway through the hydrolysis of its membrane-bound substrate, PIP_2_ [3,7]. The importance of this sperm-specific protein in human fertilization has been highlighted by the substantial evidence provided by the numerous clinical and genetic studies during the last 16 years that directly linked defects and/or deficiencies in human PLCζ with documented cases of male factor infertility [9,10,11,12,13,14]. Furthermore, findings from two separate studies that described PLCζ “knockout” mouse phenotypes confirmed the critical role of PLCζ at egg activation and monospermic fertilization in mice [31,32]. More specifically, despite the fact that both studies revealed that male mice could still produce offspring, albeit with significantly smaller litter sizes, both studies showed that sperm lacking functional PLCζ protein fail to trigger Ca^2+^ release when microinjected into mouse eggs by intracytoplasmic sperm injection (ICSI). However, in vitro fertilization (IVF) with PLCζ “knockout” sperm resulted in irregular and delayed Ca^2+^ oscillation patterns (non-physiological), along with a high degree of polyspermy and activation failure [31,32]. The atypical and delayed pattern of Ca^2+^ release, together with the decreased number of embryos and offspring, might be attributed to spontaneous activation, which is sometimes observed in certain mouse strains, when PLCζ knockout sperm was introduced. In addition, even if this is the case for mouse oocytes, it may not apply to humans, as mouse oocytes are much more likely to show Ca^2+^ oscillations and they are more sensitive to IP_3_R-induced Ca^2+^ release than human oocytes because they have higher ATP levels [15,33]. However, these studies indeed raise the possibility that there could be an alternative “primitive” or “cryptic” sperm factor, which may also contribute to egg activation in mice. However, further and thorough investigation is needed before drawing any conclusions, as it is well established that Ca^2+^ release is an integral component of egg activation in all species studied to date.

As mentioned above, the crucial role of PLCζ in human fertilization is reinforced by the continuous discovery of mutations in the PLCζ gene among patients with oocyte activation deficiencies, which lead to fertilization failure. In 2008, Yoon et al. [9] first identified a number of infertile male patients presenting with OAF due to the complete absence or reduced amounts of PLCζ in their sperm. Then, the first PLCζ mutation was reported in a non-globozoospermic patient within the Y catalytic domain (H398P) [10]. Since then, more than 20 mutations have been identified in the PLCζ gene. Among these, the majority of the point mutations are located within the catalytic X and Y domains (R197H, L224P, H233L, L246F, L277P, S350P, A384V, H398, P420L, K448N), four in the C2 domain (I489F, S500L, R553P, M578T) and one within the EF-hands (I120M) of PLCζ [3,16]. In addition to the point mutations, four frameshift mutations identified in the PLCζ gene of infertile patients (T324fs and V326Kfs∗25, located at the X-Y linker region, and N377fs and R412fs, located at the Y catalytic domain) [16]. The fact that male infertility-linked point mutations have been identified in other areas than the X and Y catalytic domains of PLCζ highlight the vital role of EF hand and C2 domains on the unique function of this sperm-specific protein. It is possible that these domains, in addition to their roles in Ca^2+^ sensitivity and lipid binding [26,34], might play an important role facilitating the interaction of PLCζ with an unknown “egg factor” which might regulate or even assist with the targeting of PLCζ to PIP_2_ stores within the intracellular vesicles, instead of the PIP_2_ stores in the plasma membrane [35]. This might be the reason why PLCζ is inactive and unable to generate Ca^2+^ release within somatic cells [36].

The progressive identification of PLCζ mutations in patients with sperm that fail to activate oocytes after ICSI resulting in total fertilization failure necessitates an effective treatment. Dai et al. reported that patients with mutations in PLCζ who had failed ICSI could be rescued by artificial oocyte activation (AOA) using the Ca^2+^ ionophore ionomycin [13]. To date, Ca^2+^ ionophores like ionomycin and calcimycin are exclusively used by the majority of IVF clinics to overcome cases of OAF after ICSI. However, these agents cause a single Ca^2+^ rise rather than the physiological pattern of Ca^2+^ oscillations. Furthermore, the safety of these ionophores is poorly studied and there is a risk that they can be associated with cytotoxic, teratogenic effects and congenital defects [16,20]. Strontium chloride is another chemical that has been used recently for AOA in patients with fertilization failure. However, Storey et al. suggested that strontium chloride fails to induce Ca^2+^ release and activate human oocytes due to their lower cytosolic ATP levels [37]. Furthermore, Lu et al. reported similar findings where strontium was unable to induce Ca^2+^ release and activate human oocytes [38]. We have previously demonstrated that injection of recombinant PLCζ protein can rescue mouse oocytes with failed activation due to a defective PLCζ protein, resulting in successful embryogenesis [27]. This study supports the notion that using the endogenous sperm protein PLCζ is the preferred “physiologic” choice to treat cases of male infertility associated with OAF. Other studies have used PLCζ RNA microinjection, which can indeed lead to oocyte activation and subsequent embryogenesis to the blastocyst stage [39]. However, a disadvantage is the unregulated and variable rate of synthesis of PLCζ protein expressed within the egg after PLCζ RNA injection. Previous studies have demonstrated that successful embryo development requires a precise PLCζ protein concentration range to produce physiological Ca^2+^ release patterns comparable to the native PLCζ amount that exists in a single mature sperm [16,21].

In the present study, we have successfully expressed and purified human MBP-tagged PLCζ recombinant protein. The MBP-tag at the N-terminus of PLCζ significantly improved the solubility of the recombinant protein within the bacterial cells and contributes to the overall protein stability after purification. MBP has been effectively used as a fusion protein with several human proteins to enhance their solubility and maintain their biological activity [40,41]. It is essential for recombinant proteins intended for therapeutic application to be stable and enzymatically active for an extended storage period. Our far-UV CD spectral analysis of MBP-PLCζ revealed a well-folded protein with a high-helical content. Heating the protein to 90 °C resulted in a significant change in the CD spectrum, indicating protein unfolding and loss of secondary structure, while the melting temperature of 60.3 °C suggests a high degree of thermal stability. A PIP_2_ hydrolysis assay was employed to investigate the enzymatic activity of the fusion protein over time, under different storage conditions. Storing the protein for extended periods of time at 4 °C and −20 °C had a progressively deleterious effect on the enzyme’s hydrolytic activity. However, cryo-storage at −80 °C was able to successfully preserve the fusion protein’s enzymatic activity for up to 90 days. Moreover, its specific enzymatic properties (EC_50_ and *Km*) were not affected. To provide a preliminary test on the safety of MBP-PLCζ, a chick embryo model was employed. The chick embryo model provides a cost-effective and reproducible method for investigating the effect of various test compounds on developmental toxicity [42]. Zero (0)-day chicken eggs were treated with a high dose of MBP-PLCζ. The Kaplan–Meier survival curve showed that exposing chicken eggs to MBP-PLCζ did not alter the embryonic viability when compared to the control, giving a first indication of safety as a potential therapeutic. However, it should be plausible in the future to use the zebrafish model to further investigate in more detail any organ-specific cytotoxic effects.

Our preclinical data support the potential use of the recombinant human MBP-PLCζ protein as a therapeutic tool for treating cases of male infertility associated with OAF. The availability of a stable, active and safe form of recombinant PLCζ protein can represent a powerful therapeutic tool for overcoming failed ICSI cases due to reduced/absent levels or mutated forms of PLCζ protein. However, further studies are necessary to investigate in more detail the therapeutic applicability of the MBP-PLCζ protein prior to its use in a clinical setting.

## Figures and Tables

**Table 1:** Recombinant human MBP-PLCζ protein retains its enzymatic properties following 90-day storage at −80 °C. The table summarizes the in vitro enzymatic properties, *K_m_* and EC_50_ values of Ca^2+^ dependence for PIP_2_ hydrolysis, determined by non-linear regression analysis (GraphPad, Prism 9.5.1; Figure 3).

Recombinant Human MBP-PLCζ Protein	K_m_ (μM)	Ca^2+^ Dependence EC_50_ (nM)
Day 1	81	57
Day 90	84	55

**Table 2:** Analysis of chicken embryos after treatment with recombinant human MBP-PLCζ protein.

Embryo	Number of Cases	Number of Embryos Died at ED7 (%)
Control	55	9 (16.4%)
Sham control	55	10 (18.2%)
Treatment	55	10 (18.2%)

## Data Availability

Data are contained within the article.
